# Supplementary material for: HER3 targeting with an antibody‐drug conjugate bypasses resistance to anti‐HER2 therapies
Source: EMBO Mol Med. 2020 Apr 24;12(5):e11498. doi: 10.15252/emmm.201911498 (PMC7207167; doi:10.15252/emmm.201911498)
Supplement: Supplementary file 1 — Appendix [file EMMM-12-e11498-s001.pdf]

HER3 targeting with an Antibody-Drug Conjugate Bypasses Resistance to anti-HER2 therapies

Lucía Gandullo-Sánchez, Emily Capone, Alberto Ocaña, Stefano Iacobelli, Gianluca Sala and Atanasio Pandiella

APPENDIX

Appendix Table S1, page 2

Appendix Table S2, page 3

Appendix Table S3, page 3

Appendix Table S4, page 4

Appendix Table S5, page 5

Appendix Table S6, page 5

Appendix Table S7, page 5

Appendix Table S8, page 6

Appendix Table S9, page 6

Appendix Table S10, page 7

Appendix Table S11, page 8

Appendix Table S12, page 9

Appendix Table S1. Antibodies used.

Antibody target	Application, dilution/amount	Catalog number	Source
p-Tyr (PY99)	Western, 1:10,000	sc-7020	Santa Cruz Biotechnol.
GAPDH	Western, 1:10,000	sc-166574	Santa Cruz Biotechnol.
Cleaved caspase 3	Western, 1:1,000	#9664	Cell Signaling Technol.
pHER2 (Tyr1221/22)	Western, 1:1,000	#2249	Cell Signaling Technol.
pHER3 (Tyr1289)	Western, 1:5,000	#4791	Cell Signaling Technol.
pHistone H3 (Ser10)	Western, 1:6,000	06-570	Merck
HER2 (Ab3)	Western, 1:10,000	OP15	Calbiochem
Calnexin	Western, 1:40,000	ADI-SPA-860-F	Enzo Life Science
BubR1	Western, 1:3,500	612503	BD Biosciences
pAKT (pS473)	Western, 1:5,000	560397	BD Biosciences
HER3	Western, 1:5,000	Made in house	Sanchez-Martin, 2012
Rabbit Ig (HRP)	Western, 1:20,000	1706515	Bio Rad Laboratories
Mouse Ig (Starbright 520)	Western, 1:5,000	12005867	Bio Rad Laboratories
Rabbit Ig (Starbright 700)	Western, 1:5,000	12004162	Bio Rad Laboratories
Human Ig (HRP)	Western, 1:40,000	109-035-088	Jackson Immunores.
Mouse Ig (HRP)	Western, 1:10,000	NA931	GE Healthcare
β-tubulin	Immunofluorescence, 1:200	T4026	Sigma-Aldrich
LAMP1	Immunofluorescence, 1:100	#9091	Cell Signaling Technol.
Rabbit Ig (Cy2)	Immunofluorescence, 1:100	111-225-144	Jackson Immunores.
Human Ig (Cy3)	Immunofluorescence, 1:800	109-165-003	Jackson Immunores.
Mouse Ig (Cy2)	Immunofluorescence, 1:100	PA42002	GE Healthcare
HER3	Immunoprecipitation, 1µg	Made in house	Sanchez-Martin, 2012
HER2	Immunoprecipitation, 1µg	Trastuzumab	Roche

Appendix Table S2. List of exact *P* values for data presented in Figure 1B.

**Figure**	**Group**	**Statistical analysis**	***P*-value**
Fig 1B	Control vs trastuzumab treatment in BT474	Two-way factorial ANOVA with Bonferroni correction	5.667x10-8
Fig 1B	Control vs trastuzumab treatment in BTRH	Two-way factorial ANOVA with Bonferroni correction	0.022
Fig 1B	Control vs trastuzumab treatment in BTRH#10	Two-way factorial ANOVA with Bonferroni correction	0.163
Fig 1B	Between all controls	Two-way factorial ANOVA with Bonferroni correction	1
Fig 1B	Trastuzumab treatment in BT474 vs trastuzumab in BRTH	Two-way factorial ANOVA with Bonferroni correction	0.000003
Fig 1B	Trastuzumab treatment in BT474 vs trastuzumab in BRTH#10	Two-way factorial ANOVA with Bonferroni correction	7.439x10-7
Fig 1B	Trastuzumab treatment in BTRH vs trastuzumab in BRTH#10	Two-way factorial ANOVA with Bonferroni correction	0.819

Appendix Table S3. List of exact *P* values for data presented in Figure 1F.

**Figure**	**Group**	**Statistical analysis**	***P*-value**
Fig 1F	Control vs EV20 in BT474	One-way ANOVA followed by Bonferroni post-hoc test	1
Fig 1F	Control vs EV20/MMAF in BT474	One-way ANOVA followed by Bonferroni post-hoc test	7.438x10-7
Fig 1F	EV20 vs EV20/MMAF in BT474	One-way ANOVA followed by Bonferroni post-hoc test	7.737x10-7
Fig 1F	Control vs EV20 in BTRH	One-way ANOVA followed by Bonferroni post-hoc test	0.290307
Fig 1F	Control vs EV20/MMAF in BTRH	One-way ANOVA followed by Bonferroni post-hoc test	3.137x10-8
Fig 1F	EV20 vs EV20/MMAF in BTRH	One-way ANOVA followed by Bonferroni post-hoc test	4.149x10-8
Fig 1F	Control vs EV20 in BTRH#10	One-way ANOVA followed by Bonferroni post-hoc test	1
Fig 1F	Control vs EV20/MMAF in BTRH#10	One-way ANOVA followed by Bonferroni post-hoc test	0.000002
Fig 1F	EV20 vs EV20/MMAF in BTRH#10	One-way ANOVA followed by Bonferroni post-hoc test	0.000002

Appendix Table S4. List of exact *P* values for data presented in Figure 2A.

**Figure**	**Group**	**Statistical analysis**	***P*-value**
Fig 2A	Control vs trastuzumab treatment in BT474	Two-way factorial ANOVA with Bonferroni correction	1.003x10-13
Fig 2A	Control vs trastuzumab treatment in BTRH	Two-way factorial ANOVA with Bonferroni correction	0.001375
Fig 2A	Control vs trastuzumab treatment in PDX118	Two-way factorial ANOVA with Bonferroni correction	3.519x10-16
Fig 2A	Control vs trastuzumab treatment in TR1	Two-way factorial ANOVA with Bonferroni correction	5.716x10-9
Fig 2A	Control vs trastuzumab treatment in TR2	Two-way factorial ANOVA with Bonferroni correction	1.712x10-9
Fig 2A	Between all controls	Two-way factorial ANOVA with Bonferroni correction	1
Fig 2A	Trastuzumab treatment in BT474 vs trastuzumab in BRTH	Two-way factorial ANOVA with Bonferroni correction	7.775x10-11
Fig 2A	Trastuzumab treatment in BT474 vs trastuzumab in PDX118	Two-way factorial ANOVA with Bonferroni correction	0.000056
Fig 2A	Trastuzumab treatment in BT474 vs trastuzumab in TR1	Two-way factorial ANOVA with Bonferroni correction	9.050x10-7
Fig 2A	Trastuzumab treatment in BT474 vs trastuzumab in TR2	Two-way factorial ANOVA with Bonferroni correction	0.000004
Fig 2A	Trastuzumab treatment in BRTH vs trastuzumab in PDX	Two-way factorial ANOVA with Bonferroni correction	9.058x10-14
Fig 2A	Trastuzumab treatment in BRTH vs trastuzumab in TR1	Two-way factorial ANOVA with Bonferroni correction	0.000083
Fig 2A	Trastuzumab treatment in BRTH vs trastuzumab in TR2	Two-way factorial ANOVA with Bonferroni correction	0.000018
Fig 2A	Trastuzumab treatment in PDX118 vs trastuzumab in TR1	Two-way factorial ANOVA with Bonferroni correction	6.209x10-11
Fig 2A	Trastuzumab treatment in PDX118 vs trastuzumab in TR2	Two-way factorial ANOVA with Bonferroni correction	1.590x10-10
Fig 2A	Trastuzumab treatment in TR1 vs trastuzumab in TR2	Two-way factorial ANOVA with Bonferroni correction	1

Appendix Table S5. List of exact *P* values for data presented in Figure 2H.

**Figure**	**Group**	**Statistical analysis**	***P*-value**
Fig 2H	ShC vs shHER3#1	One-way ANOVA followed by Bonferroni post-hoc test	0.000004
Fig 2H	shC vs shHER3#2	One-way ANOVA followed by Bonferroni post-hoc test	4.286x10-7
Fig 2H	ShHER3#1 vs shHER3#2	One-way ANOVA followed by Bonferroni post-hoc test	0.0005

Appendix Table S6. List of exact *P* values for data presented in Figure 4D.

**Figure**	**Group**	**Statistical analysis**	***P*-value**
Fig 4D	Control vs 48h EMMAF in BT474	Welch's t-test	0.002
Fig 4D	Control vs 48h EMMAF in BTRH	Welch's t-test	0.005

Appendix Table S7. List of exact *P* values for data* presented in Figure 5A.

**Figure**	**Group**	**Statistical analysis**	***P*-value**
Fig 5A	Control vs EV20/MMAF (3.3 mg/kg)	One-way ANOVA with Welch correction followed by Games-Howell post-hoc test	0.039
Fig 5A	Control vs EV20/MMAF (10 mg/kg)	One-way ANOVA with Welch correction followed by Games-Howell post-hoc test	0.034
Fig 5A	Control vs EV20	One-way ANOVA with Welch correction followed by Games-Howell post-hoc test	1
Fig 5A	EV20 vs EV20/MMAF (3.3 mg/kg)	One-way ANOVA with Welch correction followed by Games-Howell post-hoc test	0.010
Fig 5A	EV20 vs EV20/MMAF (10 mg/kg)	One-way ANOVA with Welch correction followed by Games-Howell post-hoc test	0.008
Fig 5A	EV20/MMAF (3.3 mg/kg) vs EV20/MMAF (10 mg/kg)	One-way ANOVA with Welch correction followed by Games-Howell post-hoc test	0.482

*The statistical analysis presented in this table was done for the last measured points.

Appendix Table S8. List of exact *P* values for data* presented in Figure 5B.

**Figure**	**Group**	**Statistical analysis**	***P*-value**
Fig 5B	Control vs trastuzumab	One-way ANOVA followed by Bonferroni post-hoc test	1
Fig 5B	Control vs EV20/MMAF	One-way ANOVA followed by Bonferroni post-hoc test	0.000003
Fig 5B	Control vs EV20	One-way ANOVA followed by Bonferroni post-hoc test	0.272
Fig 5B	EV20 vs EV20/MMAF	One-way ANOVA followed by Bonferroni post-hoc test	6.817x10-7
Fig 5B	EV20 vs trastuzumab	One-way ANOVA followed by Bonferroni post-hoc test	1
Fig 5B	Trastuzumab vs EV20/MMAF	One-way ANOVA followed by Bonferroni post-hoc test	3.451x10-7

*The statistical analysis presented in this table was done for the last measured points.

Appendix Table S9. List of exact *P* values for data* presented in Figure 5C.

**Figure**	**Group**	**Statistical analysis**	***P*-value**
Fig 5C	Control vs trastuzumab	One-way ANOVA followed by Bonferroni post-hoc test	1
Fig 5C	Control vs EV20/MMAF	One-way ANOVA followed by Bonferroni post-hoc test	0.000007
Fig 5C	Control vs EV20	One-way ANOVA followed by Bonferroni post-hoc test	0.018
Fig 5C	EV20 vs EV20/MMAF	One-way ANOVA followed by Bonferroni post-hoc test	0.003
Fig 5C	EV20 vs trastuzumab	One-way ANOVA followed by Bonferroni post-hoc test	0.010
Fig 5C	Trastuzumab vs EV20/MMAF	One-way ANOVA followed by Bonferroni post-hoc test	0.000005

*The statistical analysis presented in this table was done for the last measured points.

Appendix Table S10. List of exact *P* values for data presented in Figure EV1E.

**Figure**	**Group**	**Statistical analysis**	***P*-value**
Fig EV1E	Control vs EV20 in SKBR3	One-way ANOVA followed by Bonferroni post-hoc test	0.007259
Fig EV1E	Control vs EV20/MMAF in SKBR3	One-way ANOVA followed by Bonferroni post-hoc test	6.573x10-7
Fig EV1E	EV20 vs EV20/MMAF in SKBR3	One-way ANOVA followed by Bonferroni post-hoc test	0.000002
Fig EV1E	Control vs EV20 in BT474	One-way ANOVA followed by Bonferroni post-hoc test	1
Fig EV1E	Control vs EV20/MMAF in BT474	One-way ANOVA followed by Bonferroni post-hoc test	0.000004
Fig EV1E	EV20 vs EV20/MMAF in BT474	One-way ANOVA followed by Bonferroni post-hoc test	0.000644
Fig EV1E	Control vs EV20 in BTRH	One-way ANOVA followed by Bonferroni post-hoc test	1
Fig EV1E	Control vs EV20/MMAF in BTRH	One-way ANOVA followed by Bonferroni post-hoc test	0.000003
Fig EV1E	EV20 vs EV20/MMAF in BTRH	One-way ANOVA followed by Bonferroni post-hoc test	0.000002
Fig EV1E	Control vs EV20 in MDA-MB-361	One-way ANOVA followed by Bonferroni post-hoc test	0.949374
Fig EV1E	Control vs EV20/MMAF in MDA-MB-361	One-way ANOVA followed by Bonferroni post-hoc test	2.091x10-7
Fig EV1E	EV20 vs EV20/MMAF in MDA-MB-361	One-way ANOVA followed by Bonferroni post-hoc test	2.583x10-7
Fig EV1E	Control vs EV20 in HCC1419	One-way ANOVA followed by Bonferroni post-hoc test	1
Fig EV1E	Control vs EV20/MMAF in HCC1419	One-way ANOVA followed by Bonferroni post-hoc test	8.561x10-7
Fig EV1E	EV20 vs EV20/MMAF in HCC1419	One-way ANOVA followed by Bonferroni post-hoc test	0.000001
Fig EV1E	Control vs EV20 in HCC1569	One-way ANOVA followed by Bonferroni post-hoc test	0.04982
Fig EV1E	Control vs EV20/MMAF in HCC1569	One-way ANOVA followed by Bonferroni post-hoc test	4.234x10-7
Fig EV1E	EV20 vs EV20/MMAF in HCC1569	One-way ANOVA followed by Bonferroni post-hoc test	8.947x10-7
Fig EV1E	Control vs EV20 in HCC1954	One-way ANOVA followed by Bonferroni post-hoc test	1
Fig EV1E	Control vs EV20/MMAF in HCC1954	One-way ANOVA followed by Bonferroni post-hoc test	9.293x10-7
Fig EV1E	EV20 vs EV20/MMAF in HCC1954	One-way ANOVA followed by Bonferroni post-hoc test	9.100x10-7

Appendix Table S11. List of exact *P* values for data presented in Figure EV1H.

**Figure**	**Group**	**Statistical analysis**	***P*-value**
Fig EV1H	Control vs EV20/MMAF in BT474	One-way ANOVA with Welch correction followed by Games-Howell post-hoc test	0.015
Fig EV1H	Control vs NRG in BT474	One-way ANOVA with Welch correction followed by Games-Howell post-hoc test	0.648
Fig EV1H	Control vs NRG+EV20/MMAF in BT474	One-way ANOVA with Welch correction followed by Games-Howell post-hoc test	0.018
Fig EV1H	NRG vs EV20/MMAF in BT474	One-way ANOVA with Welch correction followed by Games-Howell post-hoc test	0.010
Fig EV1H	NRG vs NRG+EV20/MMAF in BT474	One-way ANOVA with Welch correction followed by Games-Howell post-hoc test	0.011
Fig EV1H	NRG+EV20/MMAF vs EV20/MMAF in BT474	One-way ANOVA with Welch correction followed by Games-Howell post-hoc test	0.016
Fig EV1H	Control vs EV20/MMAF in BTRH	One-way ANOVA with Welch correction followed by Games-Howell post-hoc test	0.004
Fig EV1H	Control vs NRG in BTRH	One-way ANOVA with Welch correction followed by Games-Howell post-hoc test	0.499
Fig EV1H	Control vs NRG+EV20/MMAF in BTRH	One-way ANOVA with Welch correction followed by Games-Howell post-hoc test	0.002
Fig EV1H	NRG vs EV20/MMAF in BTRH	One-way ANOVA with Welch correction followed by Games-Howell post-hoc test	0.009
Fig EV1H	NRG vs NRG+EV20/MMAF in BTRH	One-way ANOVA with Welch correction followed by Games-Howell post-hoc test	0.009
Fig EV1H	NRG+EV20/MMAF vs EV20/MMAF in BTRH	One-way ANOVA with Welch correction followed by Games-Howell post-hoc test	0.026

Appendix Table S12. List of exact *P* values for data* presented in Figure EV5D.

**Figure**	**Group**	**Statistical analysis**	***P*-value**
Fig EV5D	Control vs TDM1	One-way ANOVA with Welch correction followed by Games-Howell post-hoc test	0.114
Fig EV5D	Control vs EV20/MMAF	One-way ANOVA with Welch correction followed by Games-Howell post-hoc test	0.009
Fig EV5D	Control vs EV20	One-way ANOVA with Welch correction followed by Games-Howell post-hoc test	0.995
Fig EV5D	EV20 vs EV20/MMAF	One-way ANOVA with Welch correction followed by Games-Howell post-hoc test	0.000011
Fig EV5D	EV20 vs TDM1	One-way ANOVA with Welch correction followed by Games-Howell post-hoc test	0.077
Fig EV5D	TDM1 vs EV20/MMAF	One-way ANOVA with Welch correction followed by Games-Howell post-hoc test	0.011

*The statistical analysis presented in this table was done for the last measured points.
