# Supplementary material for: HER3 targeting with an antibody‐drug conjugate bypasses resistance to anti‐HER2 therapies
Source: EMBO Mol Med. 2020 Apr 24;12(5):e11498. doi: 10.15252/emmm.201911498 (PMC7207167; doi:10.15252/emmm.201911498)
Supplement: Supplementary file 2 — Expanded View Figures PDF [file EMMM-12-e11498-s002.pdf]

Expanded View Figures

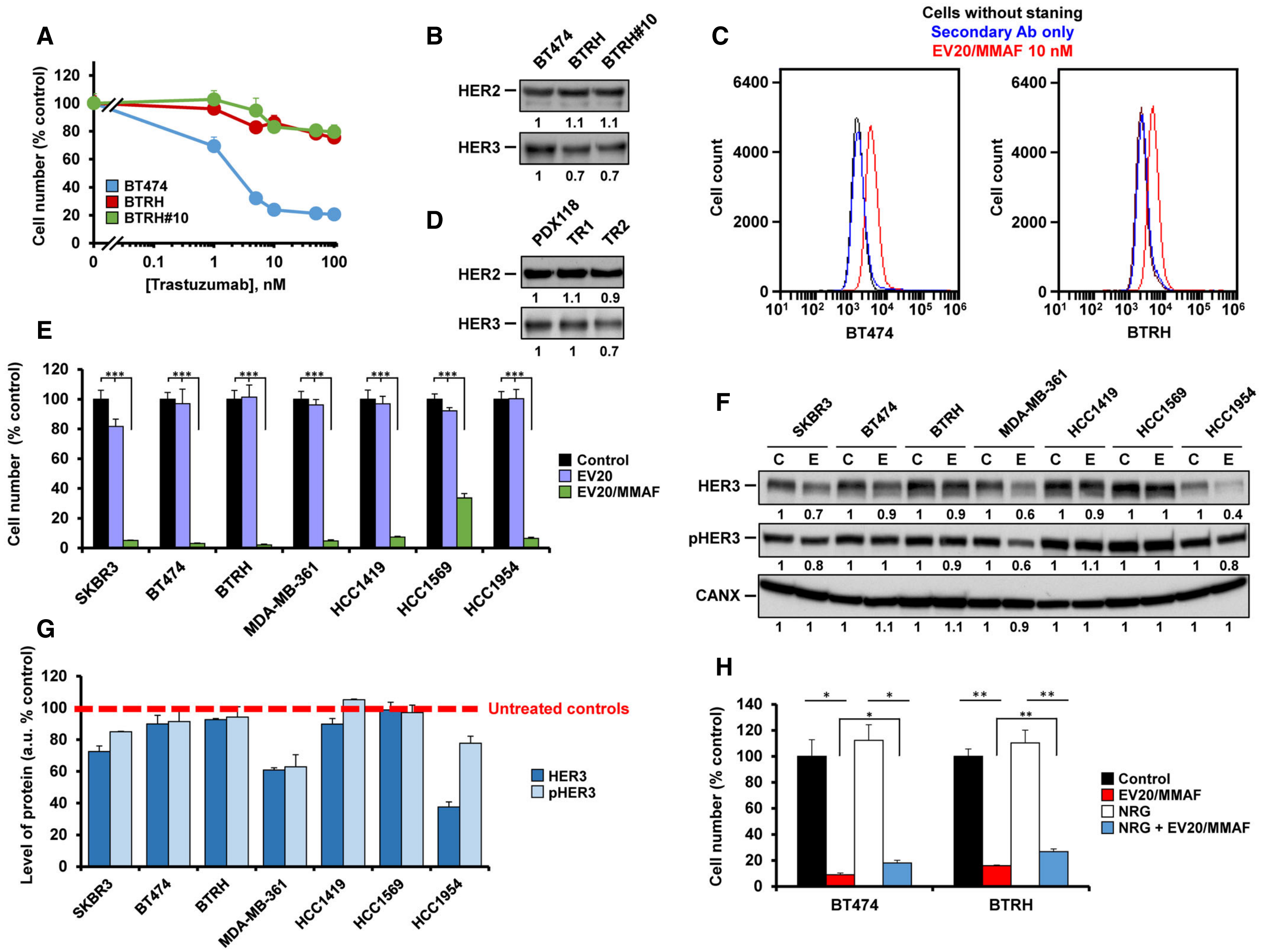

Figure EV1. Action of trastuzumab, EV20, and EV20/MMAF on different HER2⁺ cell lines.

A Dose–response analyses of the effect of trastuzumab (7 days of treatment) on the proliferation of BT474, BTRH, and BTRH#10 cells. Data represent mean + SD of triplicates of an experiment that was repeated at least four times and normalized to untreated controls. When error bars are invisible, that is because they are small and are covered by the graphic's symbols.

B Cell surface levels of HER2 and HER3 in BT474, BTRH, and BTRH#10 cells analyzed by surface immunoprecipitation.

C HER3 level was quantitatively analyzed in BT474 and BTRH cells by flow cytometry using EV20/MMAF as primary antibody and Cy3 anti-human secondary antibody.

D Cell surface levels of HER2 and HER3 in PDX118, TR1, and TR2 cells analyzed by surface immunoprecipitation.

E Action of the nude EV20 antibody or EV20/MMAF (10 nM each, 5 days) in HER2-positive breast cancer cell lines. Data represent mean + SD of triplicates of an experiment that was repeated two times and normalized to untreated controls. ***$P < 0.001$. Exact P-values of all comparisons and the statistical test used are indicated in Appendix Table S10.

F Cell lines were treated without or with EV20 (10 nM) for 5 days and lysed. HER3 (25 μg of protein) and pHER3 (60 μg of protein) were analyzed by Western, the latter with the pHER3-specific antibody.

G Quantitative analysis of the effect of EV20 on the levels of HER3 and pHER3 in each cell line, using data presented in (F) and other experiment. Intensity values of the untreated samples were taken as 100%.

H Effect of neuregulin on the action of EV20/MMAF. Cell lines were treated with EV20/MMAF (1 nM) and/or neuregulin (10 nM) for 5 days and proliferation analyzed by cell counting. Data represent mean + SD of triplicates (normalized to untreated controls) of an experiment representative of three. **$P < 0.01$. *$P < 0.05$. Exact P-values of all comparisons and the statistical test used are indicated in Appendix Table S11.

© 2020 The Authors

Figure EV2. Correlation analyses between HER3, pHER3, and sensitivity to EV20/MMAF.

A Levels of HER3 in the $HER2^+$ cell lines (BTRH, BT474, HCC1419, HCC1569, HCC1954, SKBR3, and MD-MB-361). Twenty-five μg of protein of the indicated cell lines was loaded in gels and HER3 analyzed by Western.

B Correlation analysis between IC_{50} of EV20/MMAF and HER3 levels in the $HER2^+$ cell lines. The bands corresponding to HER3 in blots shown in (A), with analogous exposure times and signals, were quantitated. Data are represented as mean ± SD of four Westerns and IC_{50} of EV20/MMAF.

C Effect of a 5 days treatment with EV20/MMAF on cell proliferation of cell lines expressing different levels of HER3 (BT474, BTRH, HS5, MDA-MB-231, and BT549). Data represent mean + SD of triplicates (normalized to untreated controls) of an experiment that was repeated at least twice.

D HER3 and pHER3 levels in the cells shown in (C). The levels of pHER3 were assessed by Western blotting of cell lysates (60 μg) using a specific pHER3 antibody.

E, F Correlation analysis between IC_{50} and pHER3 levels in the $HER2^+$ cell lines, analyzed as in (A and B). Data are represented as mean ± SD of four Westerns quantification (the first from top is the one also used for Fig 2E) and IC_{50} of EV20/MMAF.

Data information: Pearson correlation data are shown in (B and F).
Source data are available online for this figure.

© 2020 The Authors

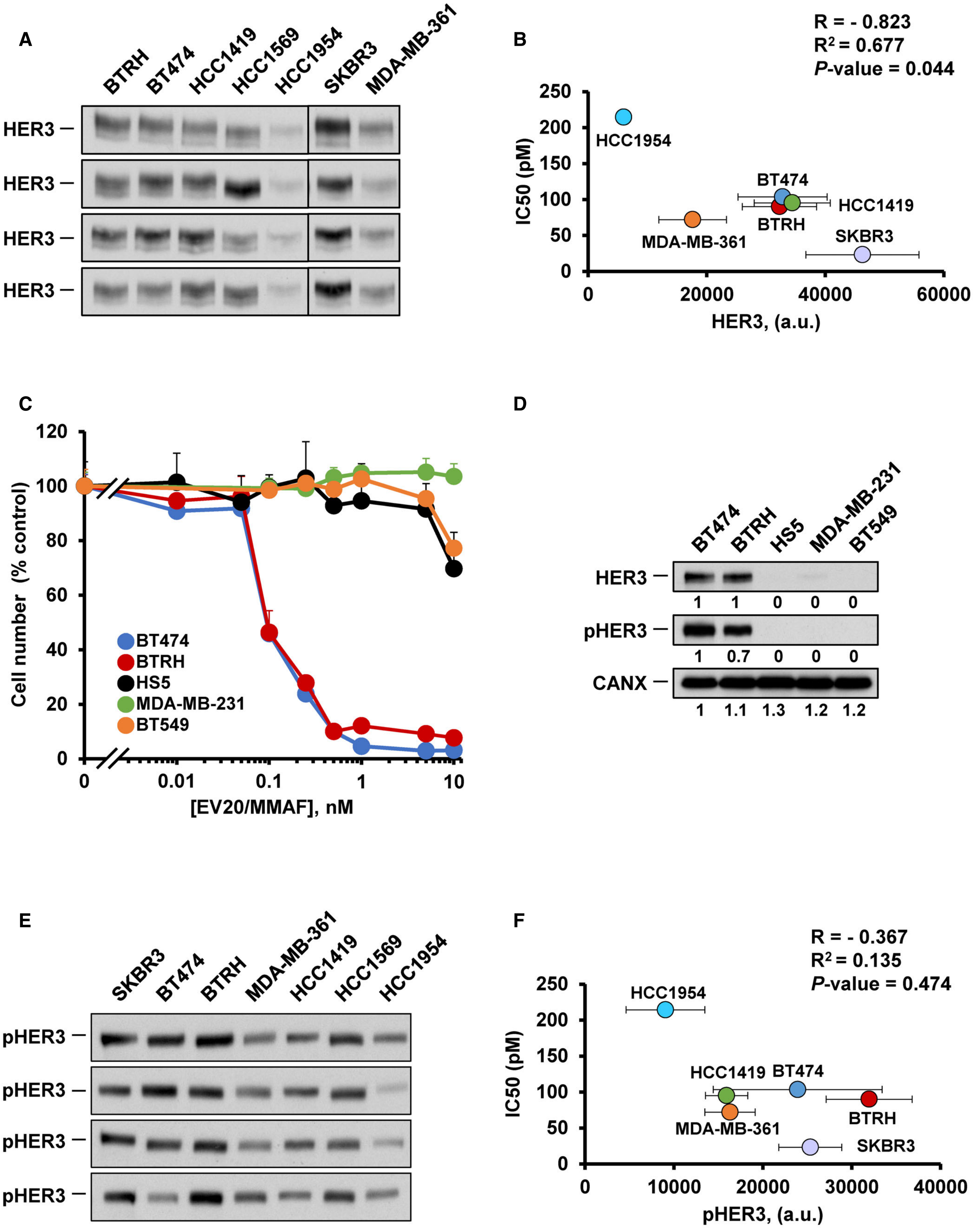
A
BTRH
BT474
HCC1419
HCC1569
HCC1954
SKBR3
MDA-MB-361
HER3
B
R = - 0.823
R^2 = 0.677
P-value = 0.044
IC50 (pM)
HER3, (a.u.)
HCC1954
BT474
HCC1419
BTRH
MDA-MB-361
SKBR3
C
Cell number (% control)
[EV20/MMAF], nM
BT474
BTRH
HS5
MDA-MB-231
BT549
D
HER3
pHER3
CANX
1 1 0 0 0
1 0.7 0 0 0
1 1.1 1.3 1.2 1.2
E
pHER3
F
R = - 0.367
R^2 = 0.135
P-value = 0.474
pHER3, (a.u.)

Figure EV2.

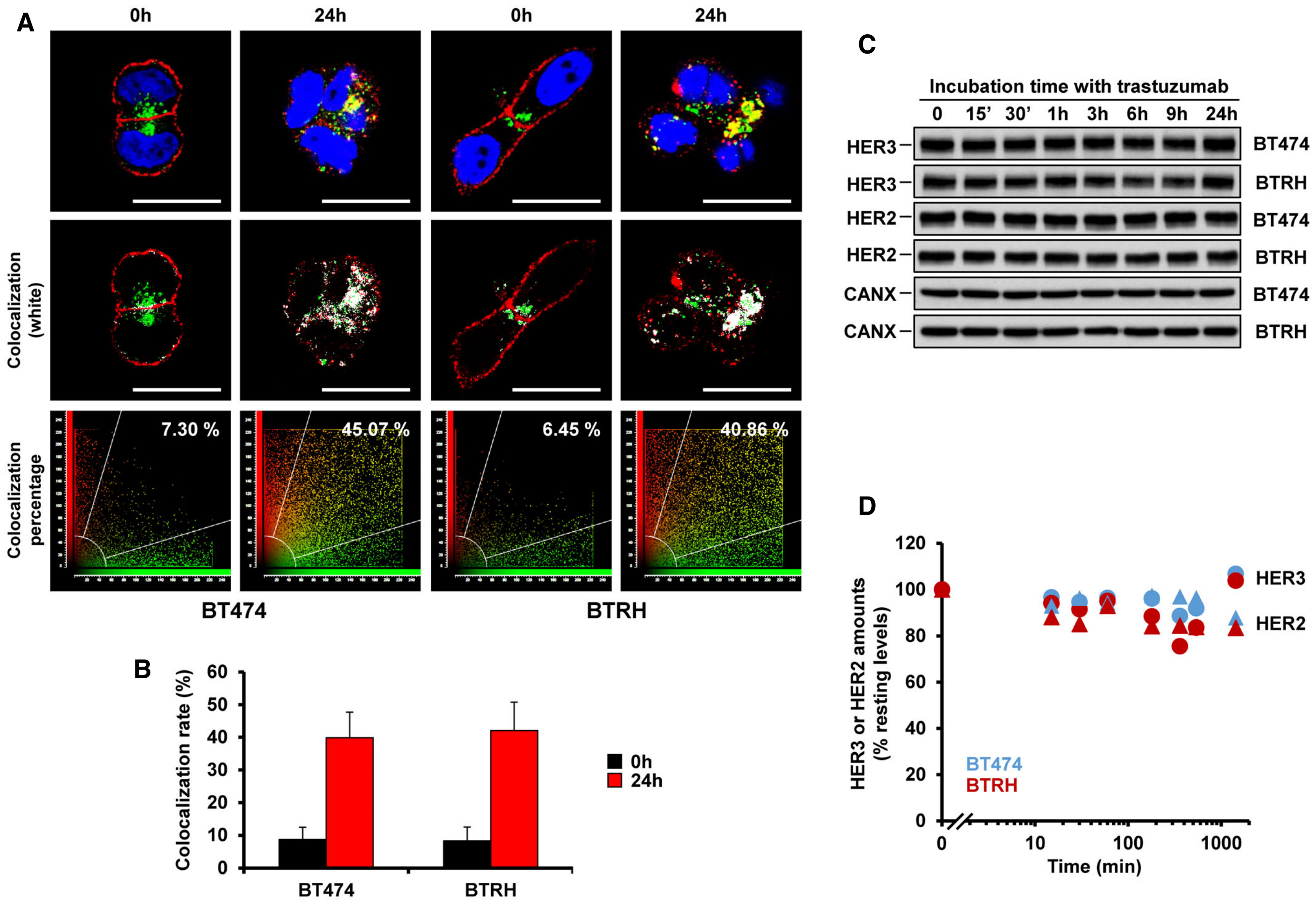

Figure EV3. Colocalization of EV20/MMAF and LAMP-1, and effect of trastuzumab on HER2 and HER3 levels in BT474 and BTRH cells.

A Colocalization of EV20/MMAF (10 nM, red) with LAMP1 (green) is shown in white (second row) in BT474 and BTRH cells. Scale bar: 20 μm. Colocalization analysis was done with Leica Application Suite Advanced Fluorescence, which generated the scatter plots of acquired images (last row). Pure red and green pixels are between abscissa/ordinate and white lines. Colocalizating pixels are found inside the central region of the plot, within the white lines.

B Quantitation of the colocalization in 20 photographs, representative of treatment with EV20/MMAF for 0 (black bars) or 24 h (red bars) in BT474 and BTRH cells. Data are represented as mean + SD.

C Western studies of the levels of HER2 or HER3 in BT474 and BTRH cells treated with trastuzumab (50 nM) for the indicated times. Lysates were prepared and equal amounts of protein (10 μg for HER2 and 25 μg for HER3) loaded in gels.

D Quantitative analyses of the experiments shown in (C).

 © 2020 The Authors

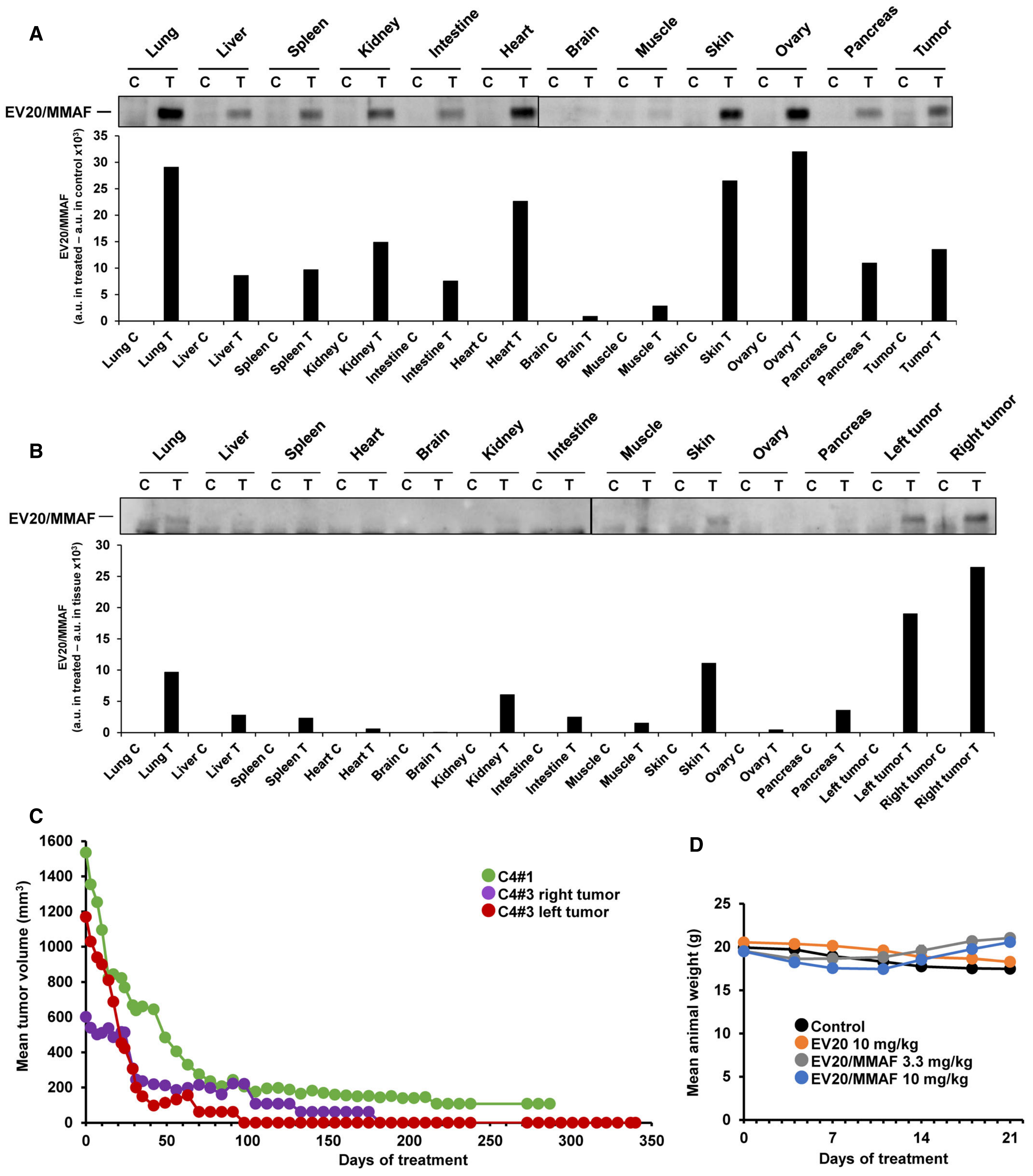
A
Lung
Liver
Spleen
Kidney
Intestine
Heart
Brain
Muscle
Skin
Ovary
Pancreas
Tumor
EV20/MMAF
EV20/MMAF (a.u. in treated – a.u. in control x10³)
B
Left tumor
Right tumor
EV20/MMAF (a.u. in treated – a.u. in tissue x10³)
C
C4#1
C4#3 right tumor
C4#3 left tumor
Mean tumor volume (mm³)
Days of treatment
D
Control
EV20 10 mg/kg
EV20/MMAF 3.3 mg/kg
EV20/MMAF 10 mg/kg
Mean animal weight (g)
Days of treatment

Figure EV4.

Figure EV4. Tissue distribution of EV20/MMAF and follow-up of tumor size in xenografted mice injected with BTRH cells.

A, B Twenty-four hours (A) or 2 weeks (B) after treatment with EV20/MMAF, tumors and indicated organs were dissected from animals. EV20/MMAF was precipitated from equal amounts of protein (500 μg) with protein A-Sepharose. EV20/MMAF was detected by Western using anti-human-HRP. The bottom part of panels (A and B) include graphical representations of the quantitation of EV20/MMAF levels shown in the Westerns, obtained as follows: signal of the band of a tissue of a treated animal minus signal of the band of the respective tissue of the untreated animal.

C Monitorization of two mice-bearing BTRH tumors treated every 3 weeks with EV20/MMAF 3.3 mg/kg.

D Mean animal weight of animals represented in Fig 5A.

Figure EV5. Response of resistant models to the drugs against which they were raised and *in vivo* evaluation of EV20/MMAF on tumor growth of a T-DM1-resistant model.

A–C Dose–response analyses of the effect of neratinib (A, 48 h, BTRN#5 and BTRN#24), lapatinib (B, 48 h, BTRL, BTRL#109, and BTRL#3), or T-DM1 (C, 5 days, BT-TDM1R#1 and BT-TDM1R#6) on the proliferation of the cells indicated in the panels. Data represent mean + SD of triplicates (normalized to untreated controls) of an experiment that was repeated at least three times. When error bars are invisible, that is because they are small and are covered by the graphic's symbols.

D *In vivo* effect of single-dose EV20 (10 mg/kg, $n = 2$), untreated control (PBS, $n = 3$), single-dose EV20/MMAF (10 mg/kg, $n = 3$), or single-dose T-DM1 (3 mg/kg, $n = 4$) on tumor growth in mice injected with BT-TDM1R#6 cells. Data are represented as mean + SEM. Exact *P*-values of all comparisons of the last measurements and the statistical test used are indicated in Appendix Table S12.

© 2020 The Authors

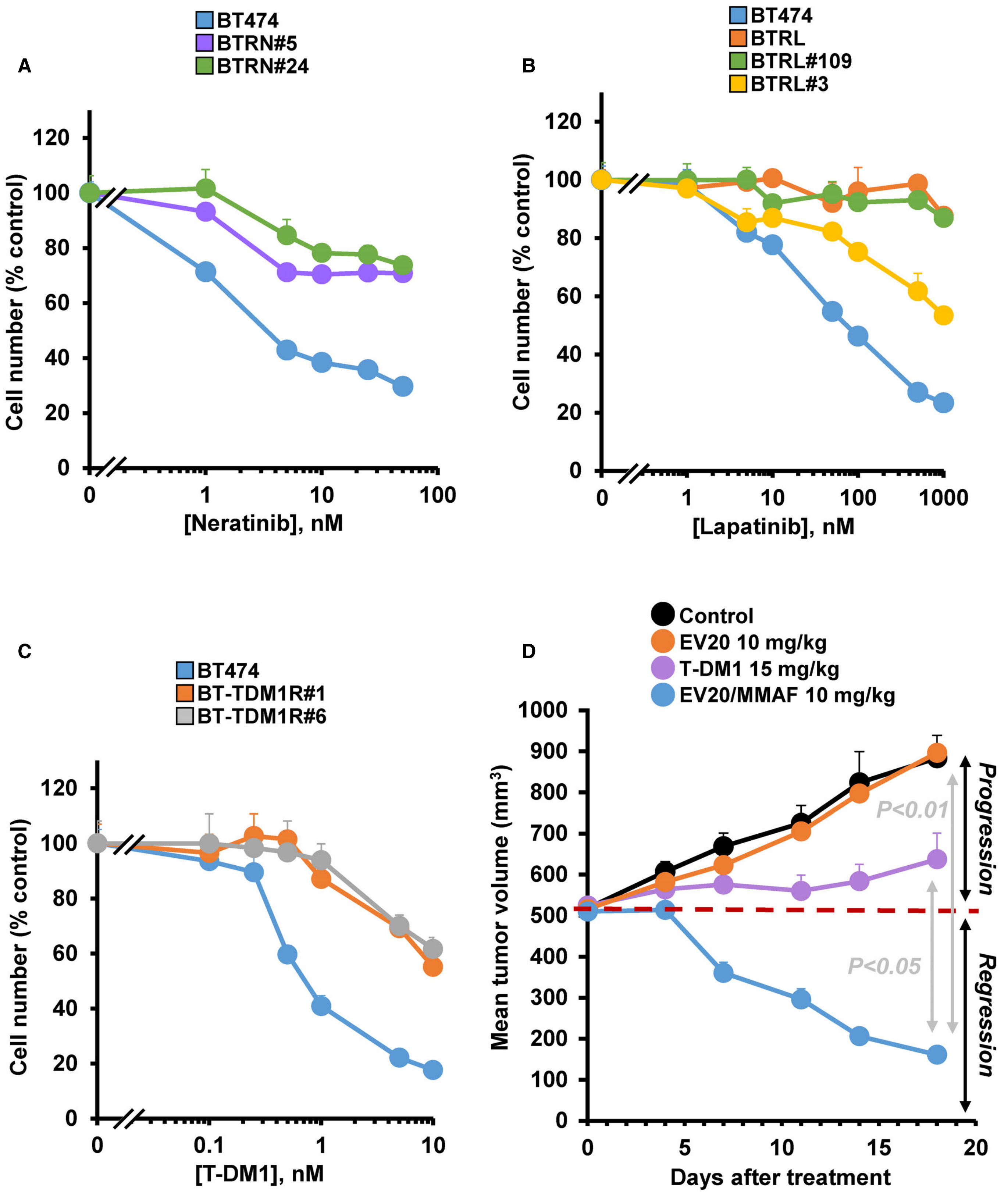
A
BT474
BTRN#5
BTRN#24
Cell number (% control)
120
100
80
60
40
20
0
0
1
10
100
[Neratinib], nM
B
BT474
BTRL
BTRL#109
BTRL#3
Cell number (% control)
120
100
80
60
40
20
0
0
1
10
100
1000
[Lapatinib], nM
C
BT474
BT-TDM1R#1
BT-TDM1R#6
Cell number (% control)
120
100
80
60
40
20
0
0
0.1
1
10
[T-DM1], nM
D
Control
EV20 10 mg/kg
T-DM1 15 mg/kg
EV20/MMAF 10 mg/kg
Mean tumor volume (mm3)
1000
900
800
700
600
500
400
300
200
100
0
0
5
10
15
20
Days after treatment
P<0.01
P<0.05
Progression
Regression

Figure EV5.
