# Supplementary material for: HER3 targeting with an antibody‐drug conjugate bypasses resistance to anti‐HER2 therapies
Source: EMBO Mol Med. 2020 Apr 24;12(5):e11498. doi: 10.15252/emmm.201911498 (PMC7207167; doi:10.15252/emmm.201911498)
Supplement: Supplementary file 4 — Movie EV2 [file EMMM-12-e11498-s004.zip › Movie_legend_EV2.docx]

**Movie legend EV2. Internalization of pHrodo-EV20/MMAF in BTRH cells.** BTRH cells were incubated with 10 nM of pHrodo-EV20/MMAF and changes in fluorescence followed along 24 hours by in vivo fluorescence microscopy. pHrodo is a red fluorogenic dye that is nonfluorescent at neutral pH and exhibits increasing red fluorescence as the pH becomes more acidic. In these cells, the intracellular fluorescence signals progressively increased along the 24 hours incubation period, indicating that pHrodo-EV20/MMAF reached acidic compartments.
