# Supplementary figures and images for: HER3 targeting with an antibody‐drug conjugate bypasses resistance to anti‐HER2 therapies
Source: EMBO Mol Med. 2020 Apr 24;12(5):e11498. doi: 10.15252/emmm.201911498 (PMC7207167; doi:10.15252/emmm.201911498)

**Figure EV2A**

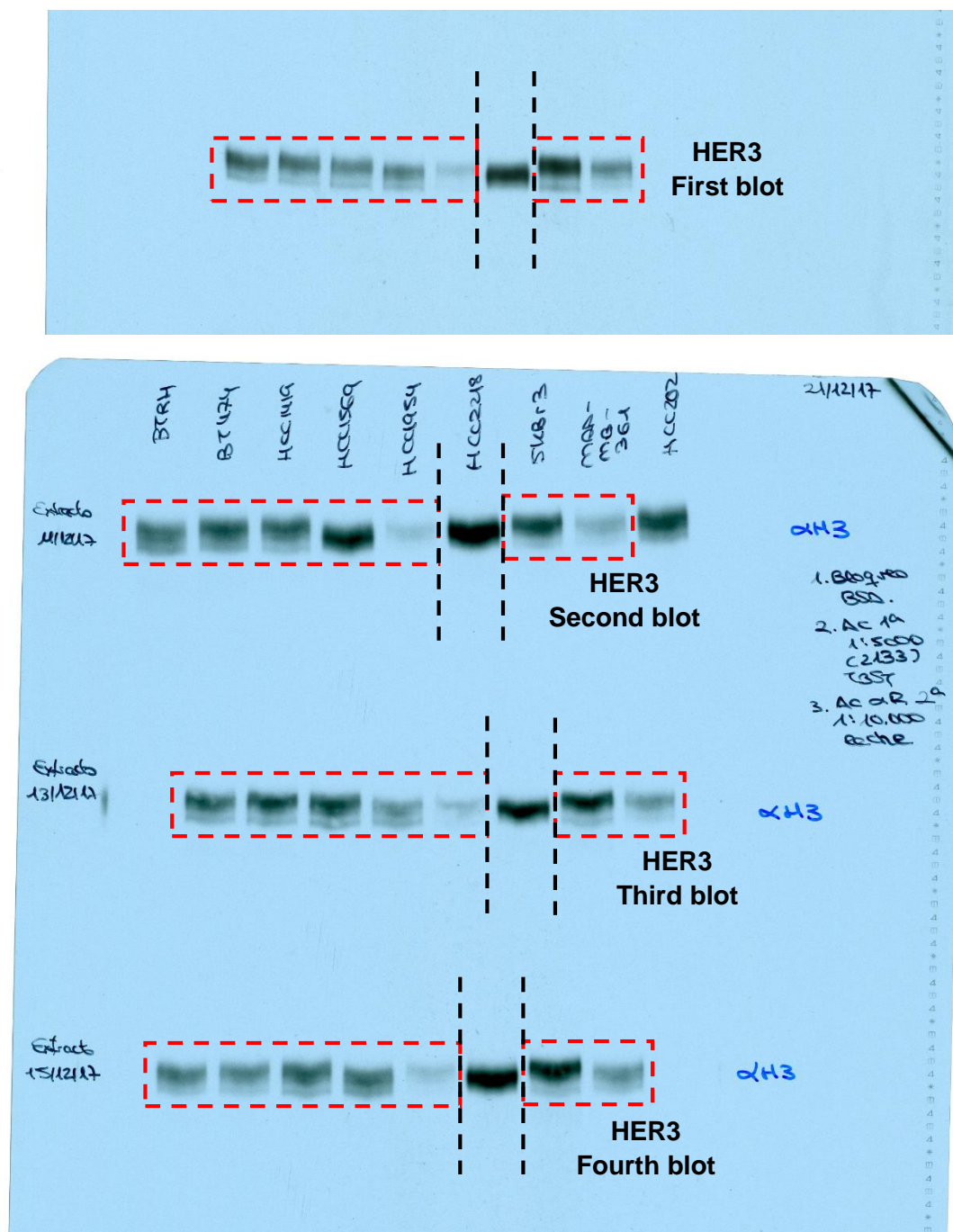

Figure EV2D

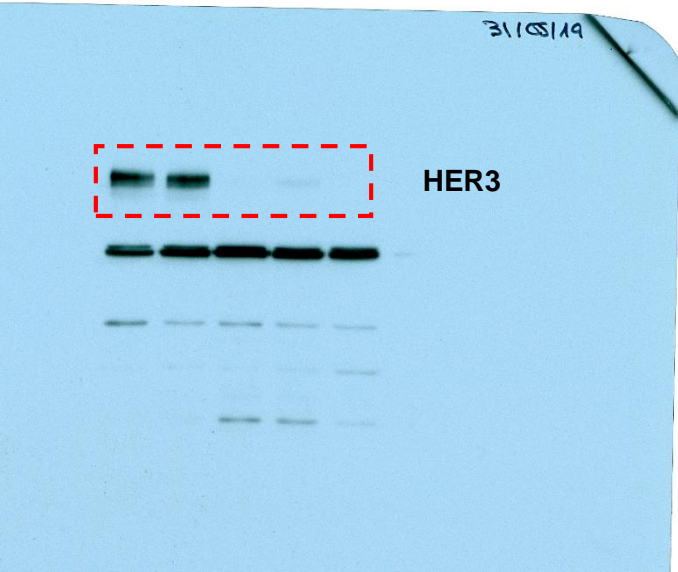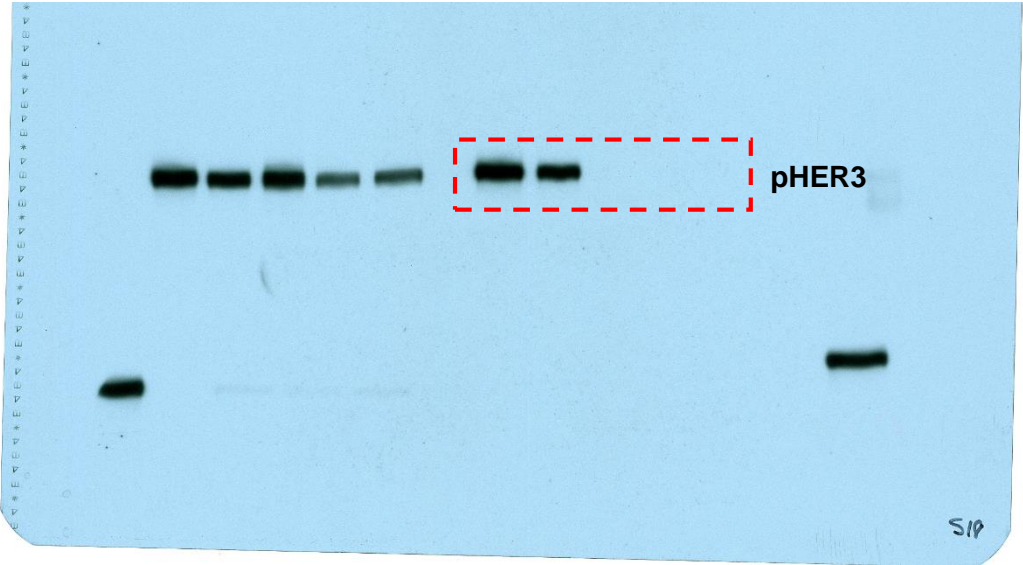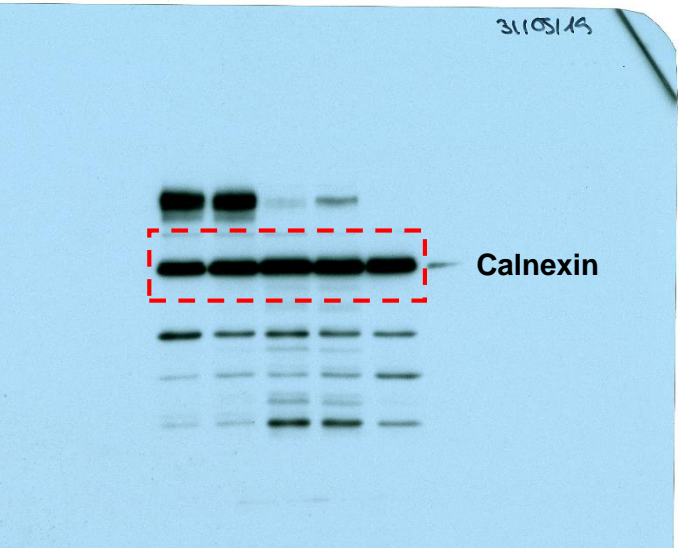

Figure EV2E

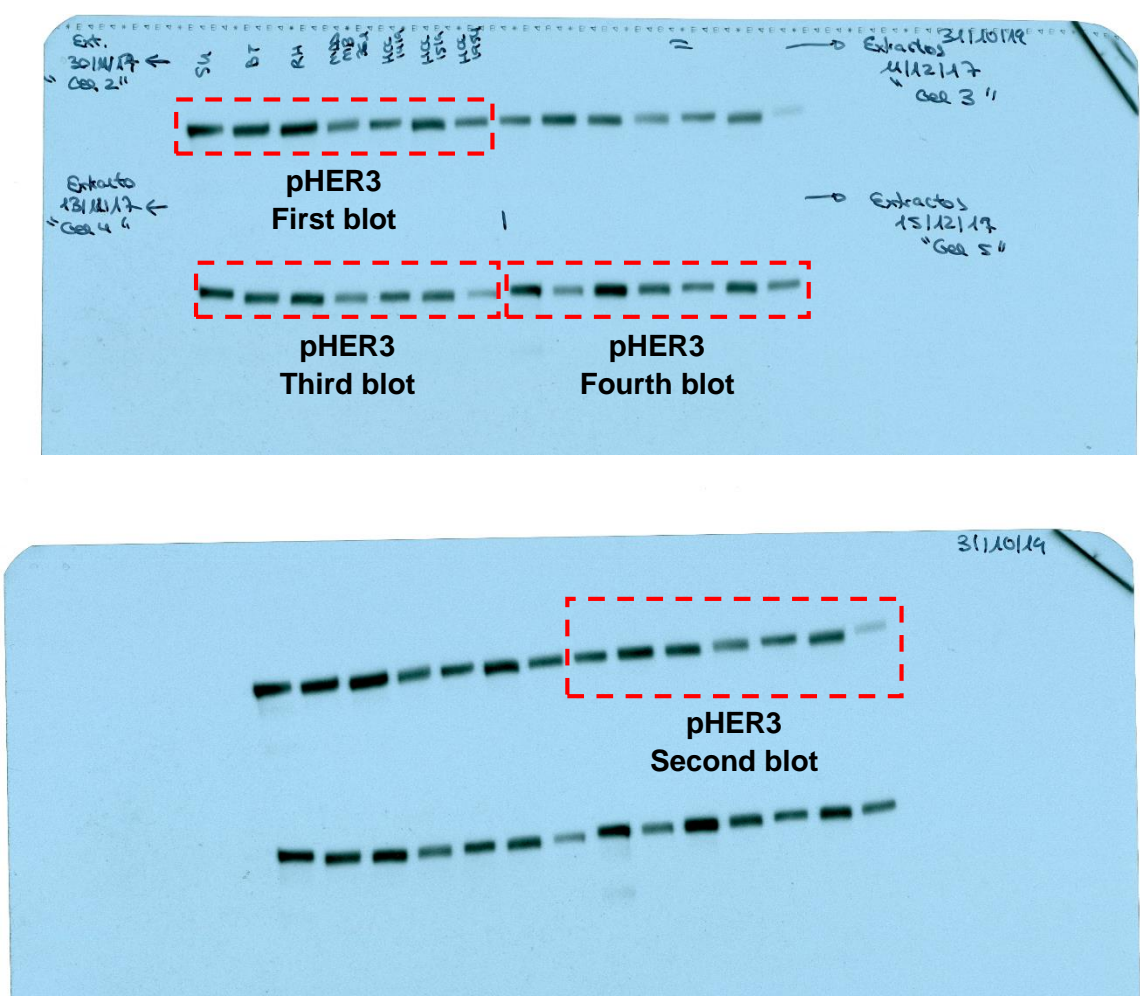

Supplement: Supplementary file 5 — Source Data for Expanded View [file EMMM-12-e11498-s007.pdf]

Figure 2B

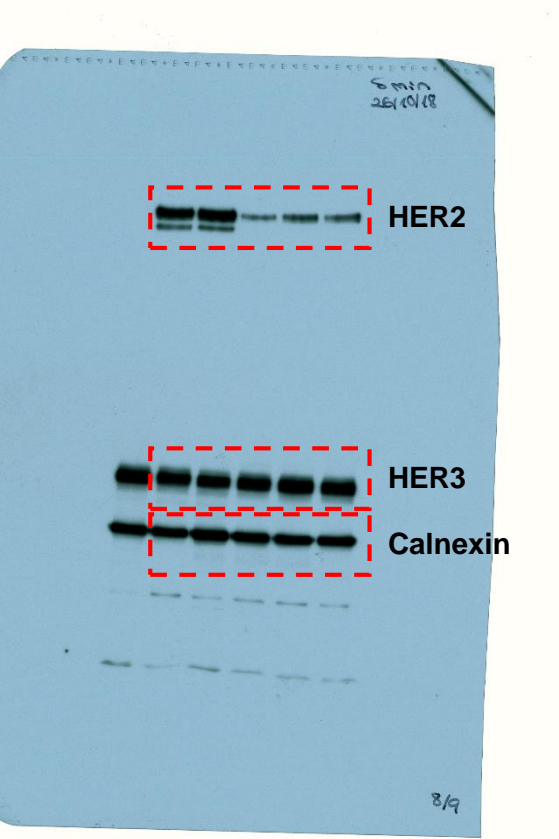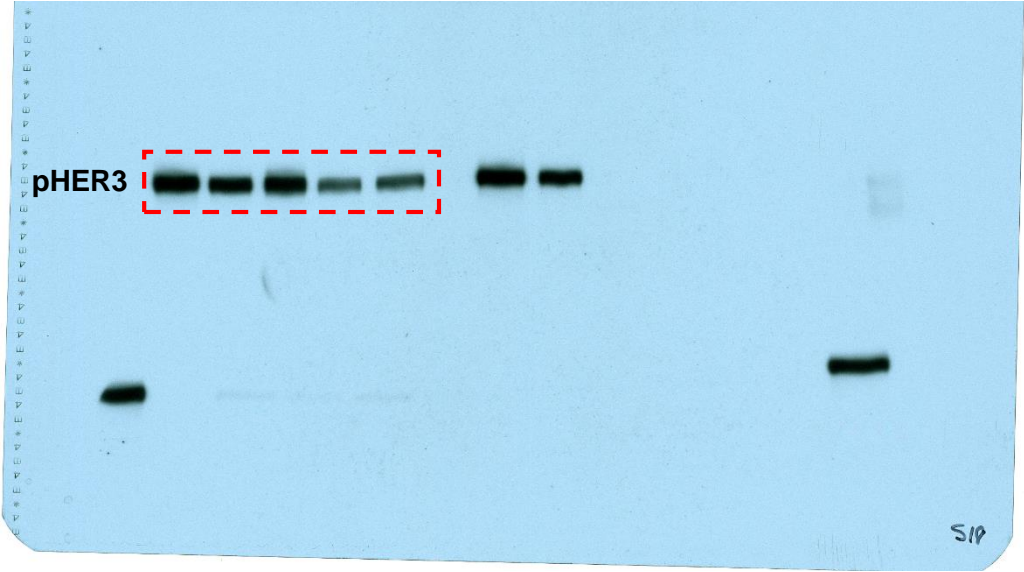

Figure 2E

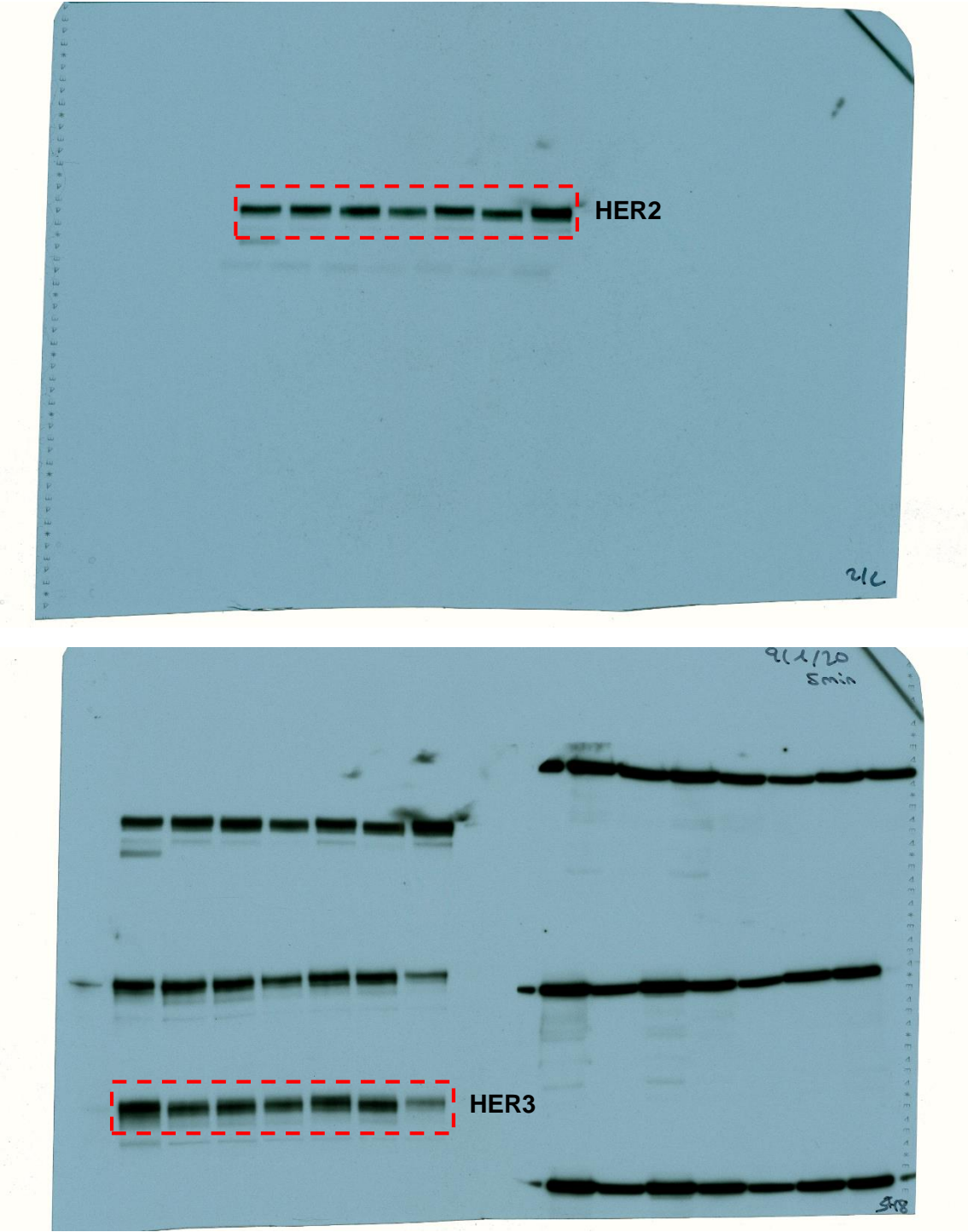

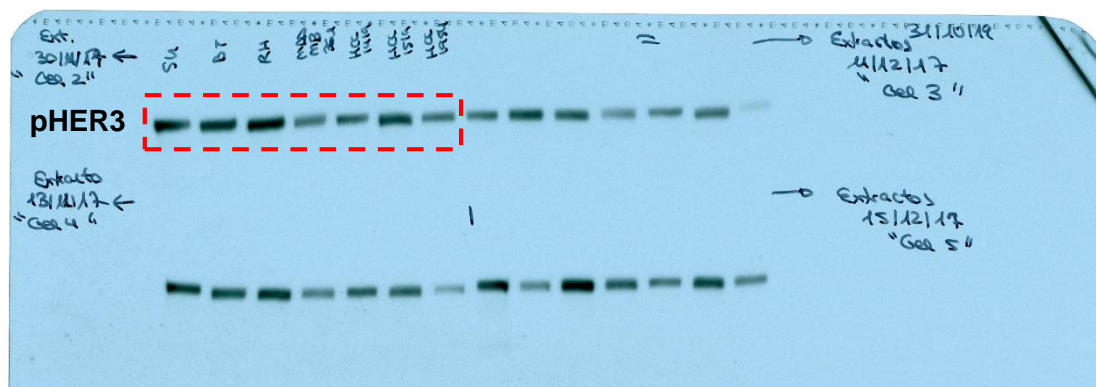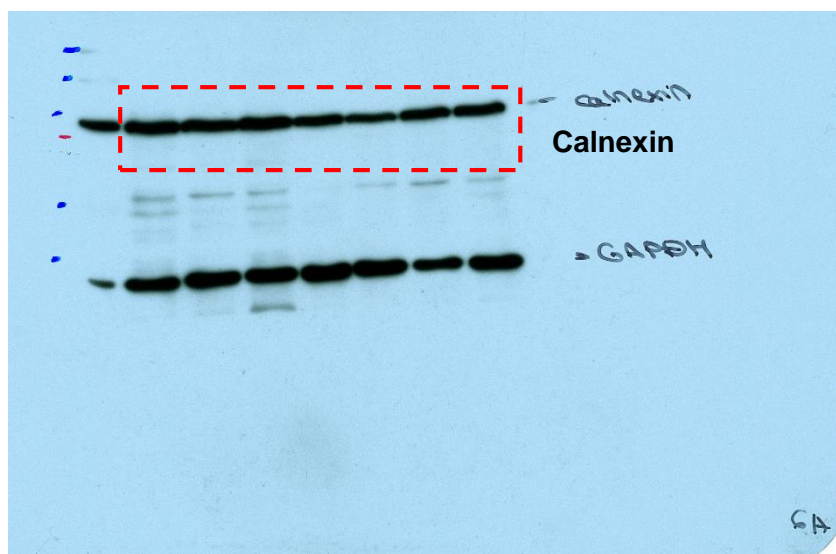

Figure 2G

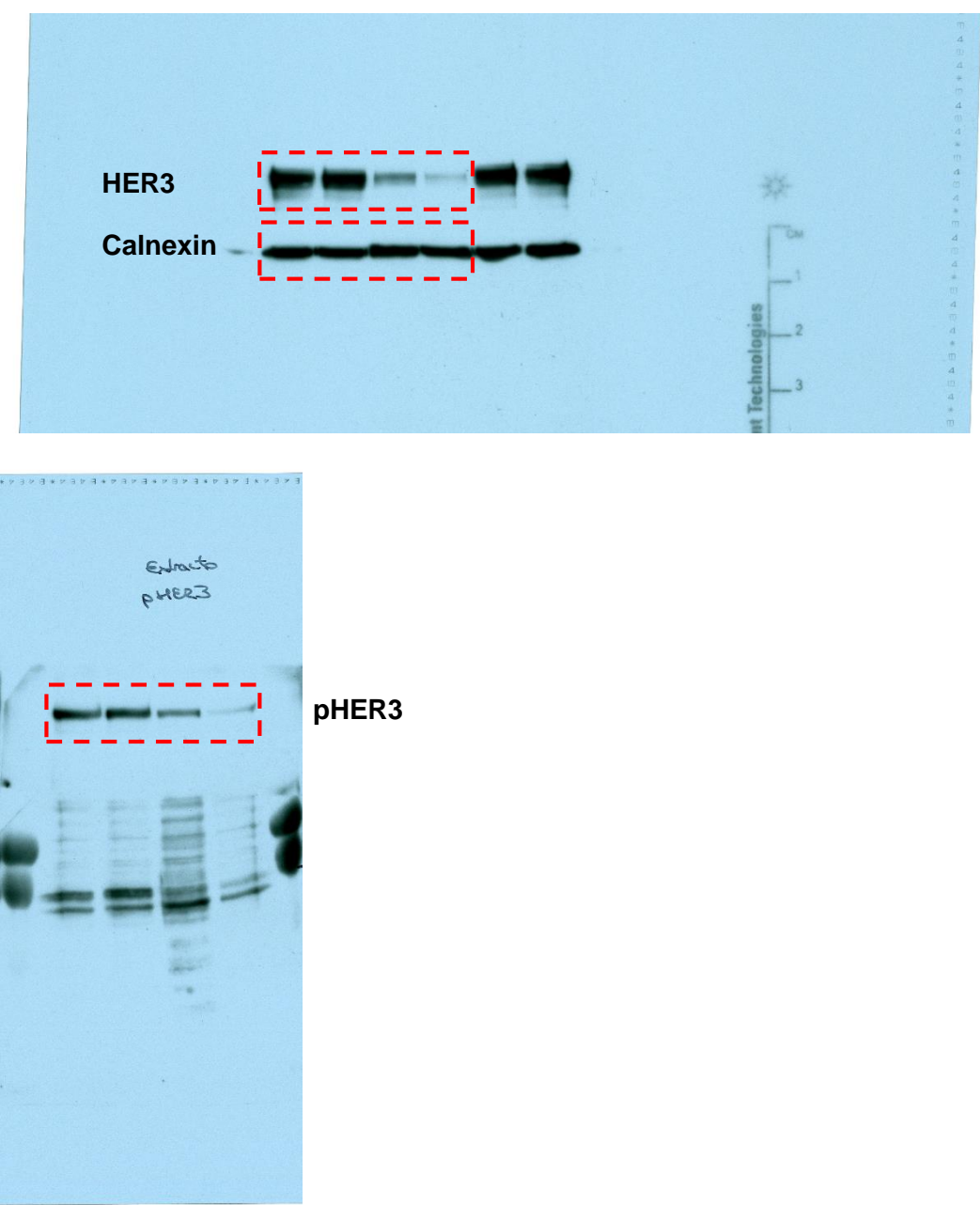

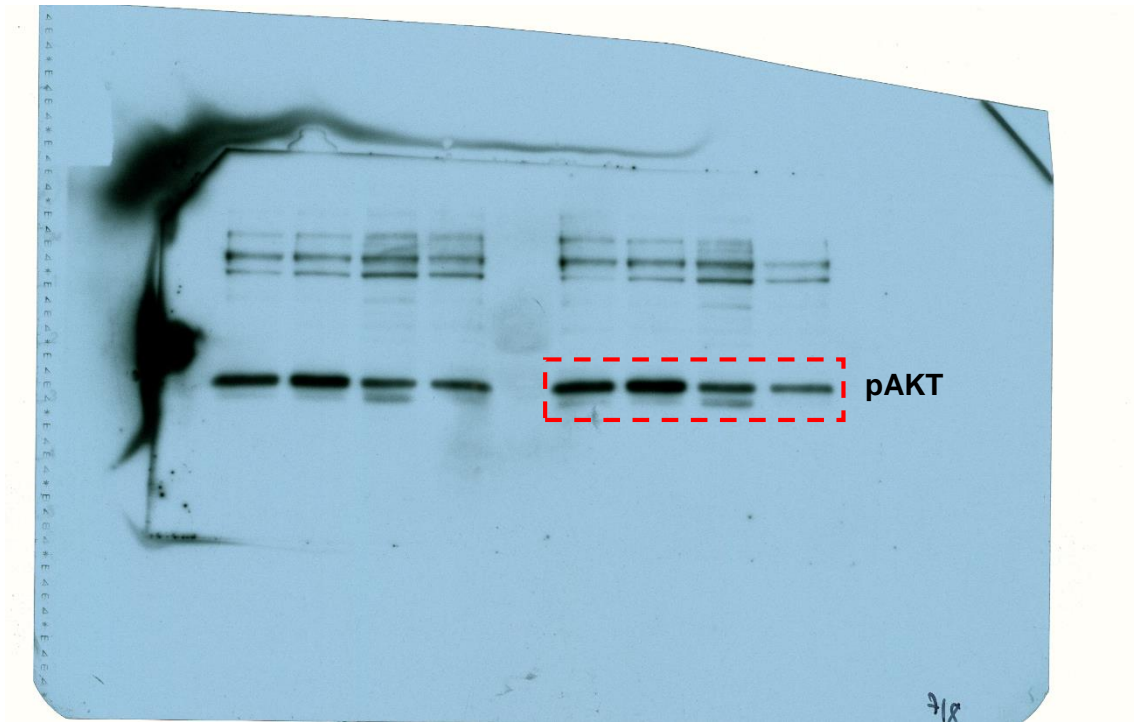

Supplement: Supplementary file 7 — Source Data for Figure 2 [file EMMM-12-e11498-s005.pdf]
