# Supplementary material for: HER3 targeting with an antibody‐drug conjugate bypasses resistance to anti‐HER2 therapies
Source: EMBO Mol Med. 2020 Apr 24;12(5):e11498. doi: 10.15252/emmm.201911498 (PMC7207167; doi:10.15252/emmm.201911498)
Supplement: Supplementary file 8 — Source Data for Figure 3 [file EMMM-12-e11498-s006.pdf]

Figure 3B

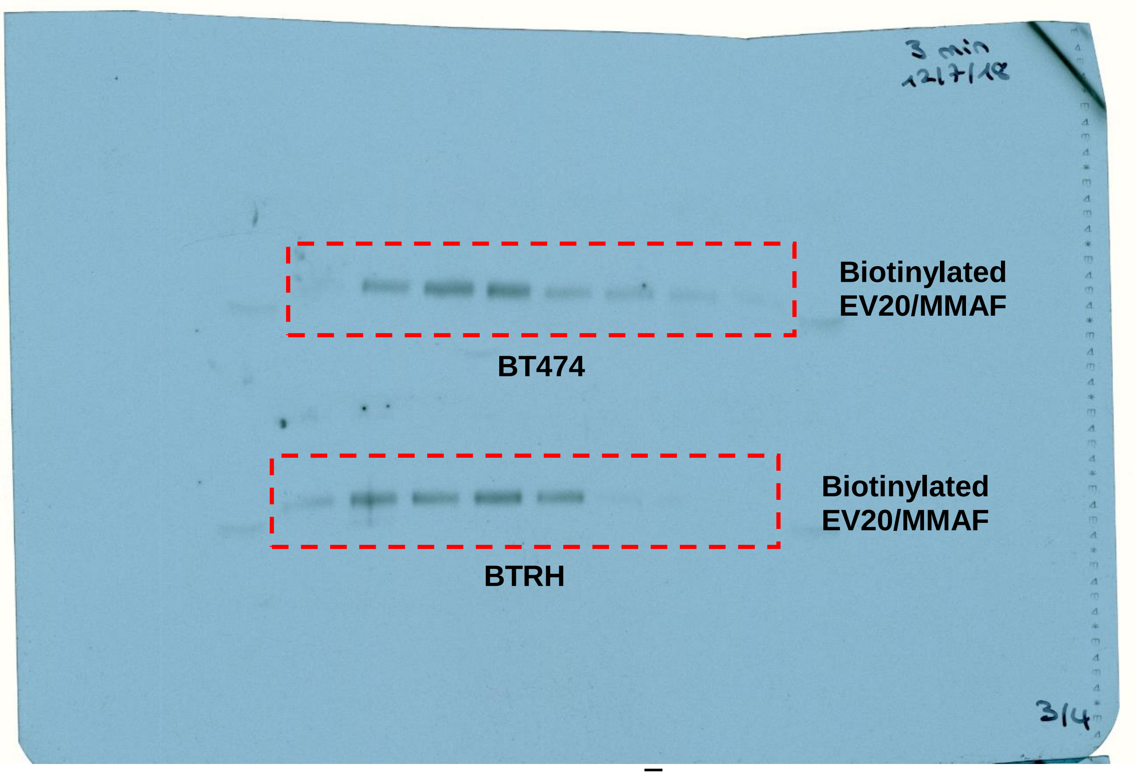

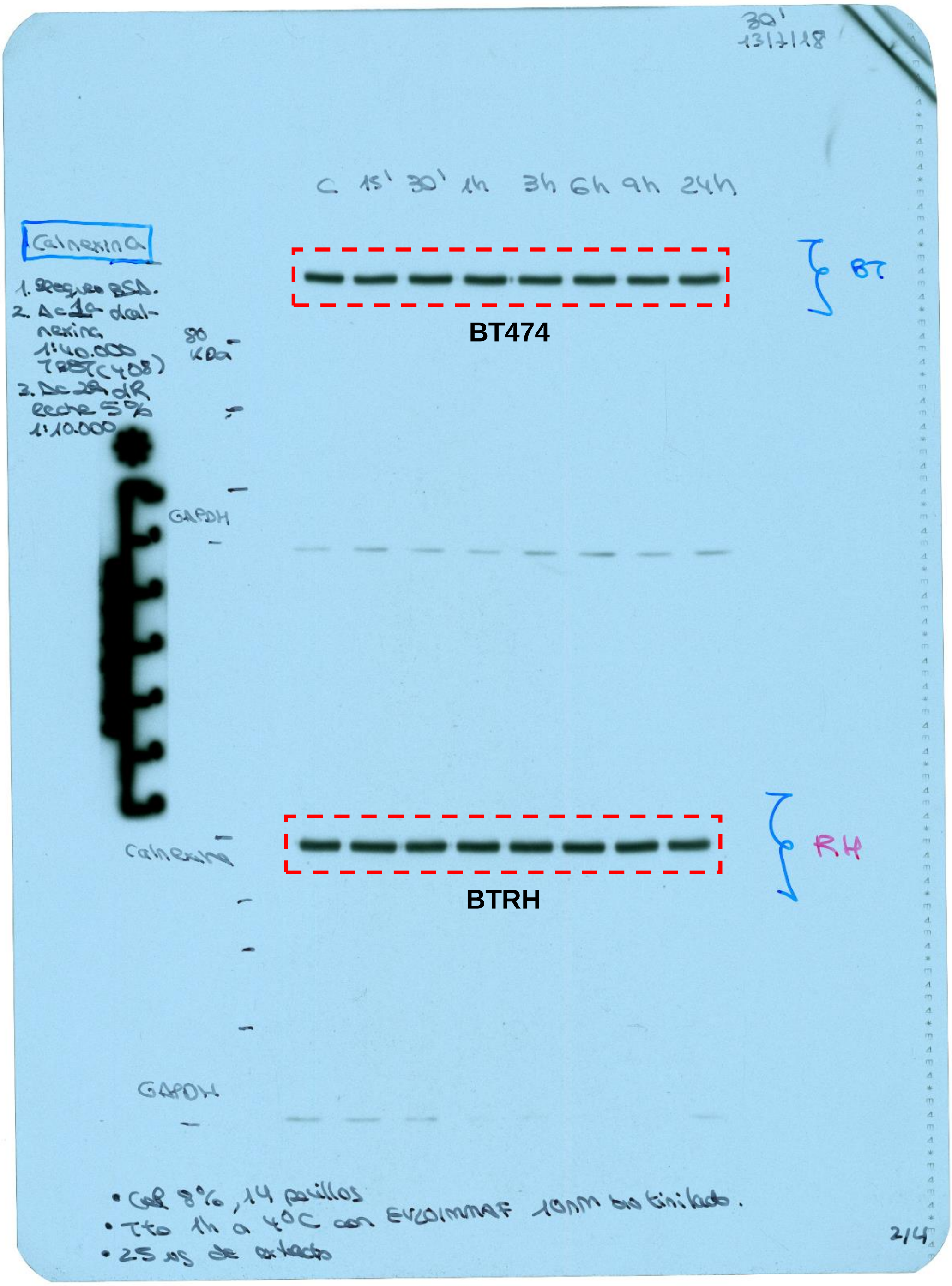
Calnexin
BT474
Calnexin
BTRH

Figure 3E

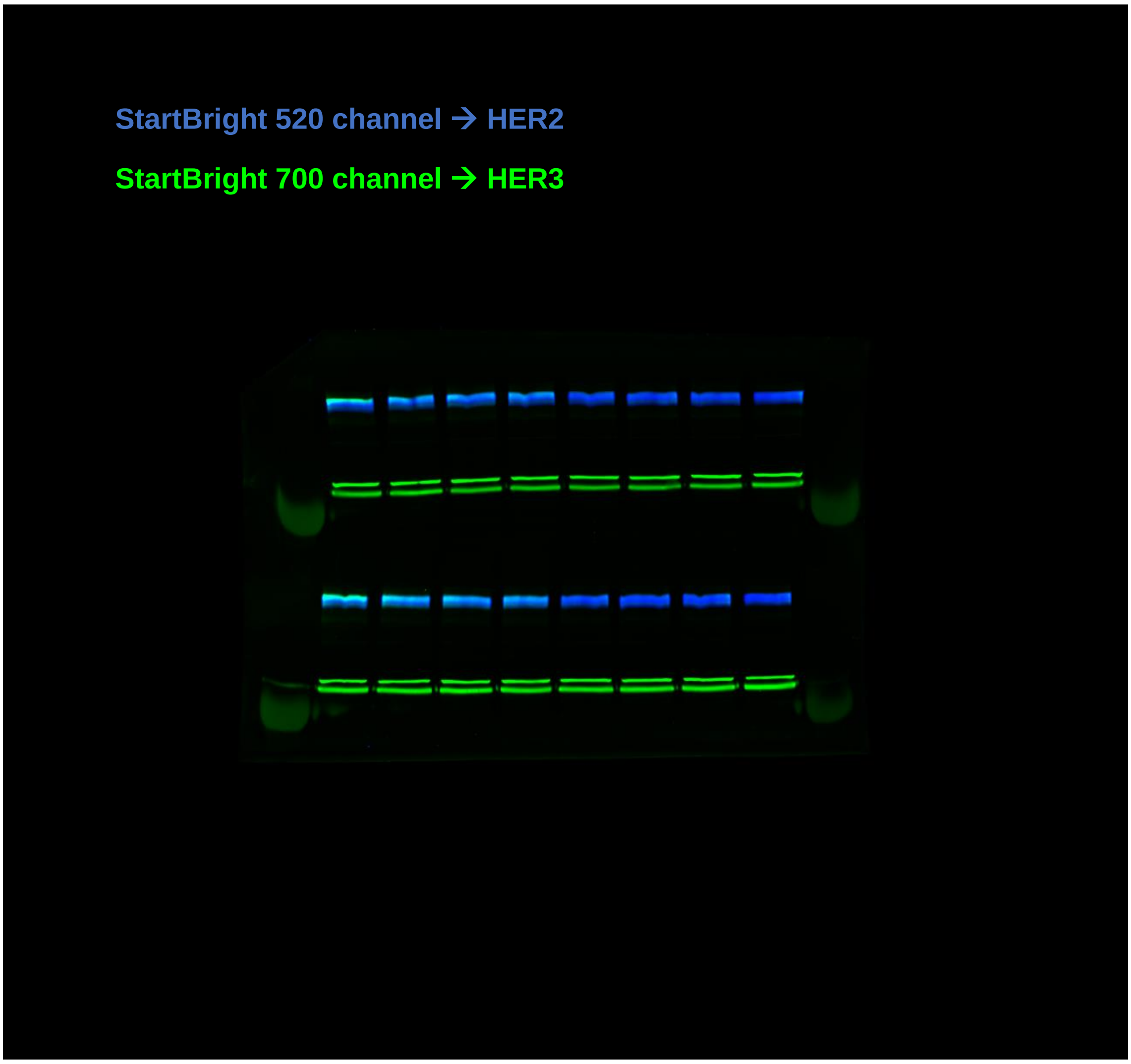
StartBright 520 channel → HER2
StartBright 700 channel → HER3

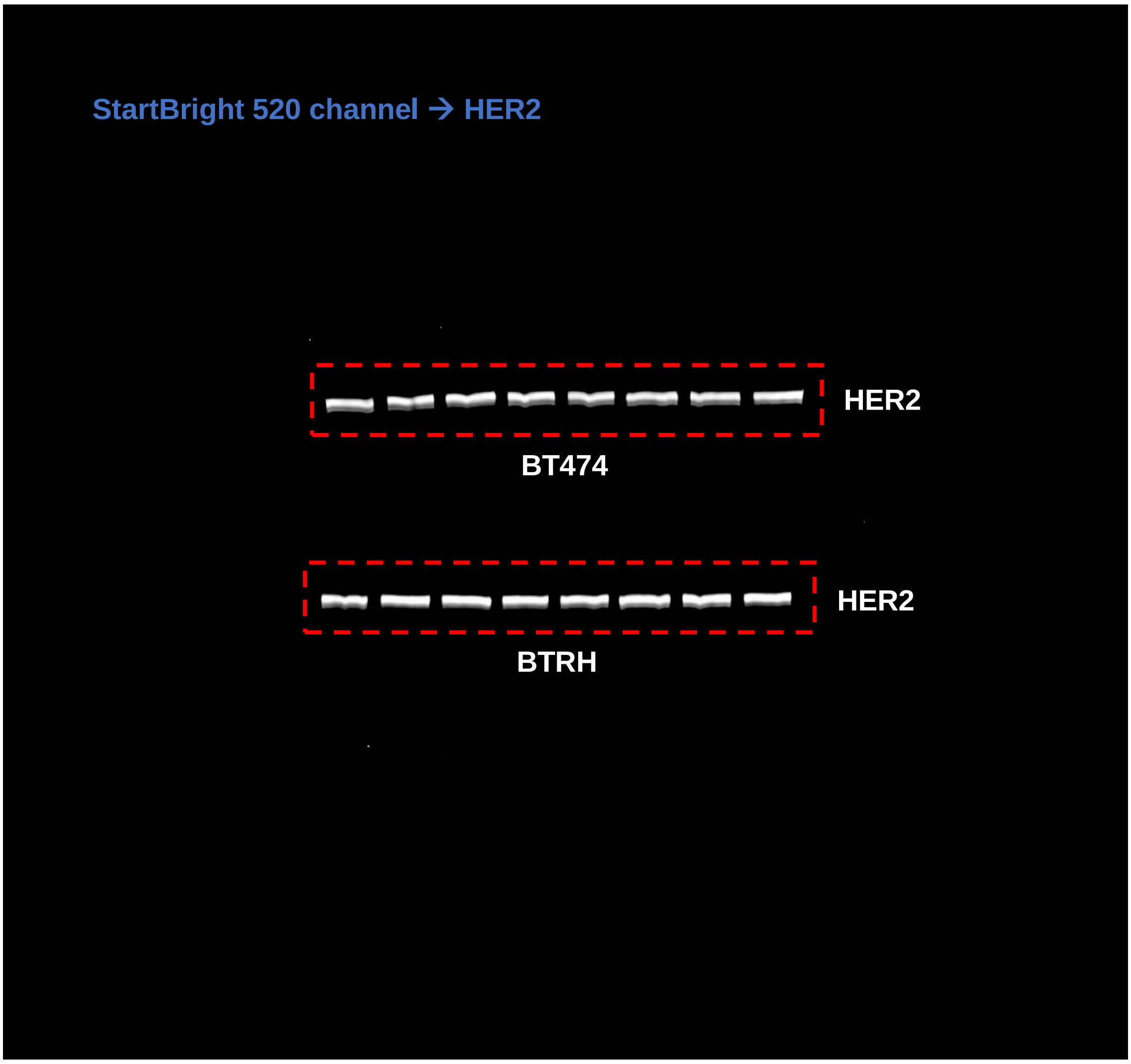
StartBright 520 channel → HER2
HER2
BT474
HER2
BTRH

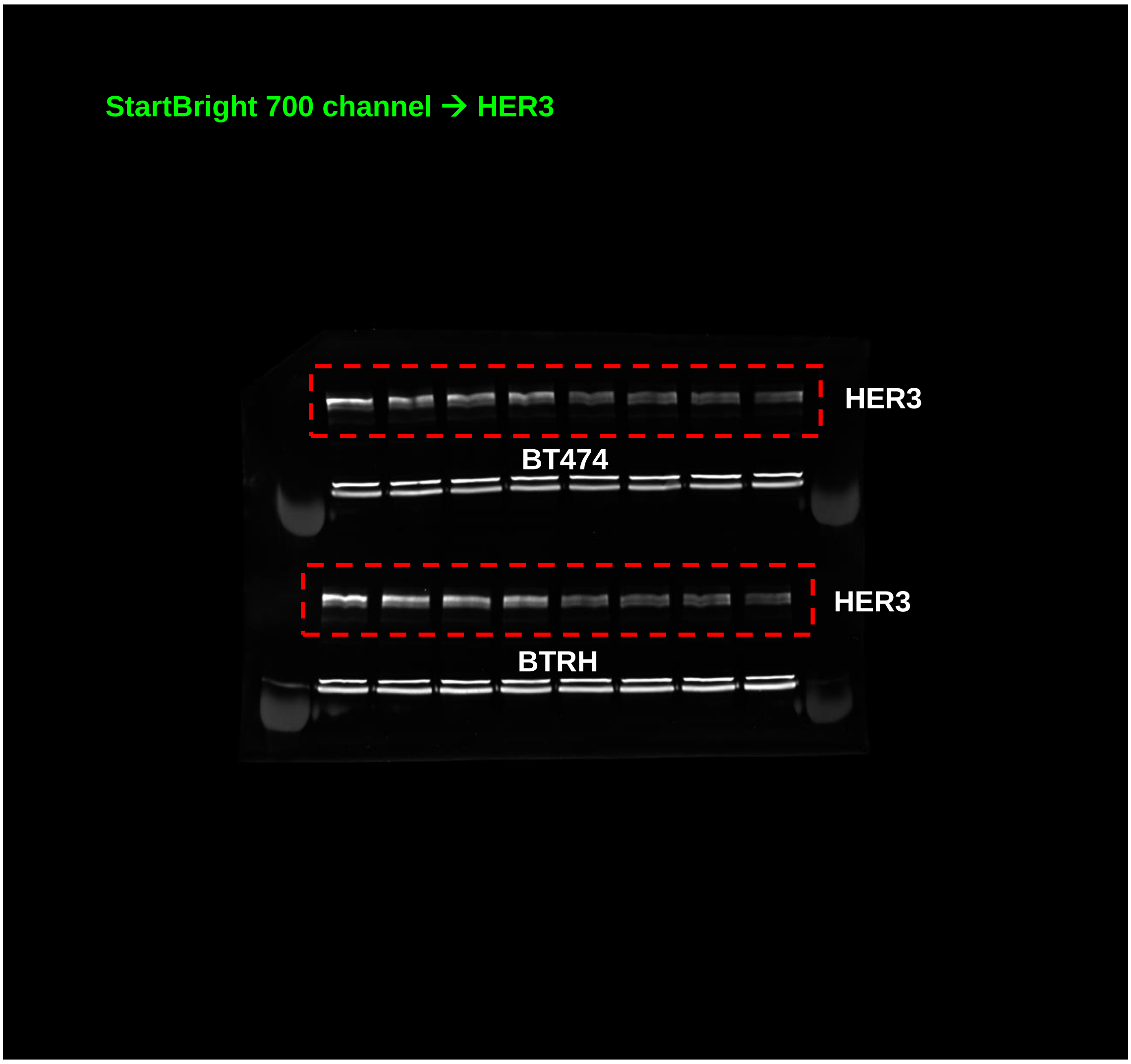
StartBright 700 channel → HER3
HER3
BT474
HER3
BTRH

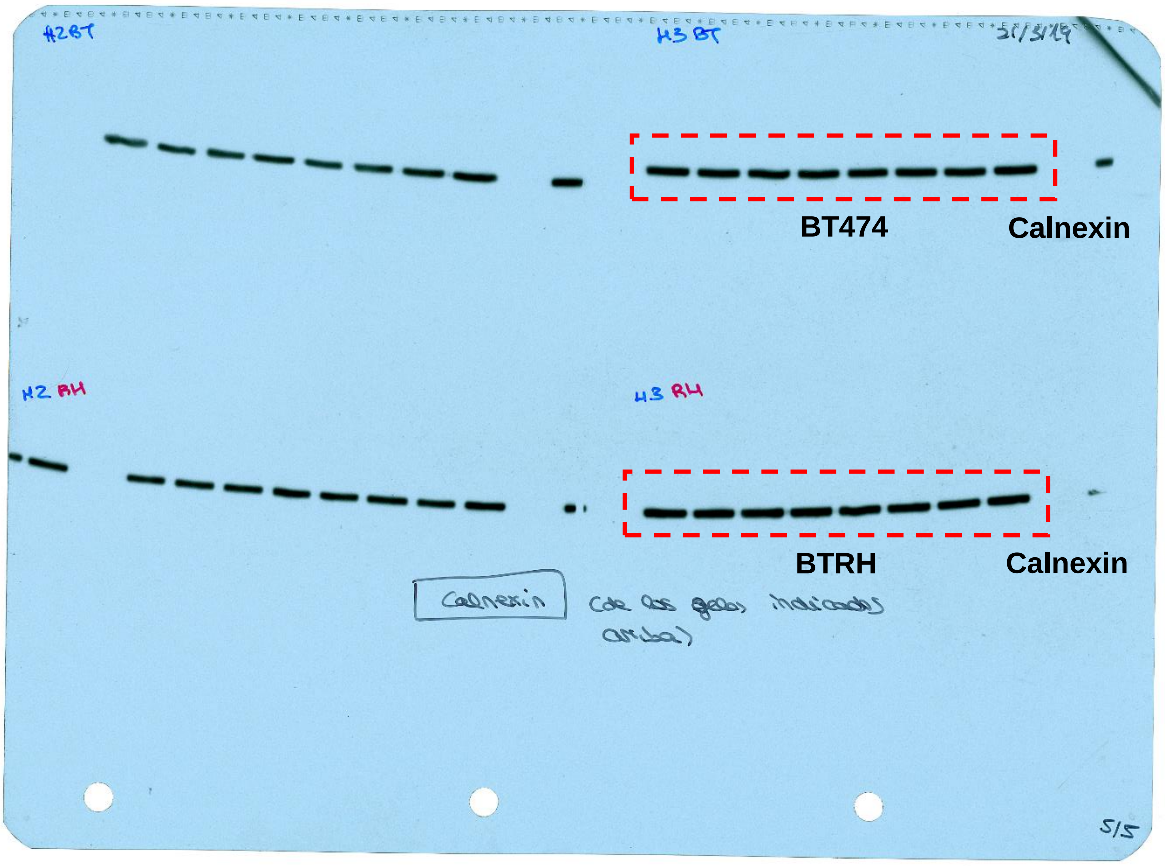
H2BT
H3 BT
BT474
Calnexin
H2 BH
H3 BH
BTRH
Calnexin
Calnexin
(de los geles indicados arriba)
5/5
